# Changes in intention to use an interprofessional approach to decision-making following training: a cluster before-and-after study

**DOI:** 10.1186/s12913-024-10899-z

**Published:** 2024-04-08

**Authors:** Hajar Taqif, Lionel Adisso, Lucas Gomes Souza, Suélène Georgina Dofara, Sergio Cortez Ghio, Louis-Paul Rivest, France Légaré

**Affiliations:** 1 Department of Mathematics and Statistics, Faculty of Science and Engineering, Université Laval, Quebec City, QC Canada; 2 VITAM - Centre de Recherche en Santé Durable, Centre Intégré Universitaire de Santé Et de Services Sociaux de La Capitale-Nationale, Quebec City, QC Canada; 3 Department of Social and Preventive Medicine, Faculty of Medicine, Université Laval, Quebec City, QC Canada; 4 Department of Family Medicine and Emergency Medicine, Faculty of Medicine, Université Laval, Quebec City, QC Canada

**Keywords:** Seniors, Home care, Health personnel, Community services, Interprofessional shared decision-making, Shared decision making

## Abstract

**Background:**

Health professionals in home care work in interprofessional teams. Yet most training in decision support assumes a one-on-one relationship with patients. We assessed the impact of an in-person training session in interprofessional shared decision-making (IP-SDM) on home care professionals’ intention to adopt this approach.

**Methods:**

We conducted a secondary analysis of a cluster stepped-wedge trial using a before-and-after study design. We collected data among home care professionals from November 2016 to February 2018 in 9 health and social services centers in Quebec, Canada. The intervention was an in-person IP-SDM training session. Intention to engage in IP-SDM pre- and post-session (dependent variable) was compared using a continuing professional development evaluation scale (CPD-Reaction) informed by the Godin’s Integrated Behavioral Model for health professionals. We also assessed socio-demographic and psychosocial variables (beliefs about capabilities, beliefs about consequences, social influence and moral norm). We performed bivariate and multivariate analysis to identify factors influencing post-intervention intention. We used the STROBE reporting guidelines for observational studies to report our results.

**Results:**

Of 134 respondents who provided complete pairs of questionnaires (pre- and post-), most were female (90.9%), mean age was 42 (± 9.3) years and 66.9% were social workers. Mean intention scores decreased from 5.84 (± 1.19) to 5.54 (± 1.35) (Mean difference = -0.30 ± 1.16; *p* = 0.02). Factors associated with higher intention post-intervention were social influence (ß = 0.34, *p* = 0.01) and belief about capabilities (ß = 0.49, *p* < 0.01).

**Conclusion:**

After in-person IP-SDM training, healthcare professionals’ intention to engage in IP-SDM decreased. However, the scope of this decrease is probably not clinically significant. Due to their association with intention, beliefs about capabilities, which translate into having a sense of self-competency in the new clinical behavior, and social influences, which translate into what important others think one should be doing, could be targets for future research aiming to implement IP-SDM in home care settings.

**Supplementary Information:**

The online version contains supplementary material available at 10.1186/s12913-024-10899-z.

## Background

Canada’s population is aging, and people aged 65 and over (older adults) are living longer than ever [1, 2]. As a society we must ensure that these older adults are supported when they need to make decisions about safe and comfortable accommodation.

Aging is generally associated with a higher risk of developing disabilities that can lead to a loss of autonomy. As they start losing their autonomy, older adults are faced with one of the most difficult decisions: to stay at home or to move somewhere where they will be able to receive appropriate care (e.g., a health care facility or a nursing home) [3]. This decision is very difficult to make alone. The shared decision-making approach (SDM) represents a middle ground between the traditional paternalistic approach, where the health care professional makes the decision alone, and the consumer approach, where the patient is the sole decision maker [4]. An interprofessional (IP) approach to SDM is especially relevant to caring for the frail elderly, as chronic illness often means that several different kinds of healthcare professional are involved in their care. The interprofessional approach to SDM (IP-SDM) enables health professionals to collaboratively support patients in facing difficult decisions and reach healthcare choices that are agreed on by the patient, their family members or caregivers, and the interprofessional team [3, 5]. Various obstacles hindering SDM have been identified, particularly within multidisciplinary care settings. These barriers include a lack of knowledge of what other disciplines can do, insufficient trust in the expertise of other disciplines, and inadequate communication among the different disciplines [6]Consequently, training home care teams in SDM through an IP approach that directly addresses these barriers is expected to facilitate the seamless adoption of decision tools in clinical practice. This, in turn, is anticipated to contribute to the widespread implementation of SDM across the health and social care system.

Based on our previous research using the IP-SDM approach [7, 8], and considering the potential of an intervention involving task-sharing among diverse kinds of professionals to alleviate the burden of the discussion about transitioning to long term care, we hypothesized that IP-SDM training would increase the intention of healthcare professionals in home care teams to engage in this approach.

To strengthen our analysis, we used Godin's integrated model, which explains healthcare professionals' clinical behavior, as the theoretical framework for our study. Godin et al. conducted a systematic review of 76 studies examining the influence of social cognitive theories on healthcare professionals' adoption of clinical behaviors [9]. The authors confirmed the key role of intention as a predictor of behavior and identified six psychosocial factors that influence intention: beliefs about capabilities and consequences, moral norms, social influences, role and identity, and individual characteristics. Based on this framework, Legare et al. developed the CPD-Reaction tool for evaluating continuing professional development courses [10]. The tool evaluates intention (using 2 of the 12 included items) immediately after training and has been shown to be a good predictor of behavior six months after the training [11]. In addition, it measures the psychosocial factors that influence intention, which suggests elements which could be target in future behavior change interventions [10, 12]. Thus this tool was useful for measuring the impact of our intervention and possible psychosocial targets to improve it.

Several studies that have measured behavioral intention used a cross-sectional design [13–15]. However, intention scores measured at a single point in time do not demonstrate a change in professionals’ intention to adopt a behavior. Therefore, our study aimed to assess the impact of IP-SDM training on healthcare professionals' intention to engage in an IP-SDM approach, both before and after an in-person training session, and to identify factors that influenced this intention.

### Methods

#### Study design

We reported this cluster pre-post study according to the STROBE reporting guidelines for observational studies [16]. We performed a secondary analysis of an existing database collected during a stepped-wedge study [17]. This primary study aimed to scale up and evaluate the implementation of SDM in interprofessional home care teams caring for older adults or their caregivers facing a decision about staying at home or moving elsewhere. The study was conducted from November 2016 to February 2018 in 9 health and social services centers (HSSCs) in the province of Quebec in Canada. HSSCs are regional health authorities that provide public health and social care for the population of their region. The trial was registered at ClinicalTrials.gov (NCT02592525) on October 30, 2015 and the protocol was published [6].

In this study, we used a pre-post measurement design with clusters to compare intention and its variables (pre and post-intervention) to engage in an IP-SDM approach among healthcare professionals in home care teams and, post-intervention, we analyzed significant factors of intention.

#### Participants

Of the 22 HSSCs contacted, 9 participated in the study. Since this was a secondary analysis of a cluster randomized trial, selection criteria applies to the site rather than the individual: all clinicians at the site were invited to participate in the training. Thus healthcare professionals from interprofessional home care teams who were involved in the care of older adults with loss of autonomy, practiced in one of the participating HSSCs, and gave informed consent (*n* = 281) were included in the study (Additional file 1).

#### Intervention

Before taking the in-person training program, healthcare professionals in home care teams were invited to complete the Ottawa Decision Support Tutorial (1h30), an online general SDM tutorial (Additional file 2) [18]. The 3.5-h in-person training, based on adult education principles, was designed according to the IP-SDM conceptual model [6]. In the context of decision-making with older adults about housing, it addressed communication techniques and strategies for engaging frail older adults with cognitive impairment or their caregivers in the decision making. It included the use of a patient decision aid (PtDA), and involved a lecture, a video, and a role play session[6].

#### Data collection and variables

Data was collected before and after each of the 9 in-person IP-SDM training sessions from November 2016 to February 2018, using the self-administered CPD-Reaction Questionnaire, which was adapted to the home care context [7]. Questionnaires were completed upon participants' arrival in the training room and at their departure. The CPD-Reaction questionnaire is a validated theory-based tool [10, 12] that followed a strict development procedure. It assesses the impact of training on clinical behavioral intention using items based on Godin’s integrated socio-cognitive model for healthcare professional behavior change [10]. CPD-Reaction evaluates behavioral intention and its psychosocial variables using 12 questions that are scored with a Likert-type scale from 1 to 7, except for one question (on social influence) which is scored on a scale of 1 to 5. The study questionnaire also collected the sociodemographic characteristics of healthcare professionals in home care teams[19].

##### Dependent variable: intention

The dependent variable of this study was the intention of healthcare professionals in home care teams to engage in an IP-SDM approach, defined as the mean of the scores of the 2 CPD-Reaction questions (items) on intention as measured on a 7-point Likert-type scale (1 = Strongly Disagree to 7 = Strongly Agree).

##### Independent variables (predictor variables)

Our independent variables were beliefs about capabilities (healthcare professional’s perceptions of facilitators and barriers to adopting the behavior, 3 items), beliefs about consequences (the usefulness and the benefits/risks of adopting the behavior, 2 items), social influence (perception of approval or disapproval by significant persons regarding the adoption of the behavior, 3 items), moral norm (feeling of personal obligation to adopt the behavior, 2 items) and sociodemographic characteristics of healthcare professionals in home care teams: number of clients served per week, age, sex, number of years of practice in home care, profession, highest level of education attained, and whether they had completed the online general SDM Tutorial and the in-person IP-SDM training session.

#### Statistical analysis

Only health professionals in home care teams who completed both questionnaires before and after attending the in-person IP-SDM training were included in the analyzes (*n* = 134).

We used descriptive statistics to report on the intention of healthcare professionals in home care teams to engage in IP-SDM and on the other 4 psychosocial variables before and after the intervention, and to describe the sociodemographic characteristics of the participants, using frequencies (n, %) for the categorical variables and mean and standard deviation (SD) for the continuous variables. For the education variable, given that participants could complete multiple response categories, we treated the variable as an ordinal of highest education obtained using the classification of the Quebec educational system.

The data we analyzed herein is from all 9 health and social services centers, making it non-independent (clustering effect).

Using repeated measures models and Wilcoxon signed ranks tests, as the normality assumption was rejected, we compared the levels of intention to engage in IP-SDM as well as the 4 psychosocial variables before and after the intervention, proceeding with the intention-to-treat analysis. This kind of analysis is appropriate for practical clinical scenarios as it makes allowance for non-compliance and protocol deviations [20]. Then we performed a sensitivity analysis by excluding participants who may or may not have been exposed to the intervention (we have no evidence of their presence). In additional analysis, we compared the pre-intervention intention of healthcare professionals who attended the preliminary online general SDM tutorial before attending the in-person training session to the pre-intervention intention of those who skipped the preliminary online tutorial, in order to see the impact of the online tutorial as well.

To determine which factors influenced participants’ intention to engage in IP-SDM, we then fit mixed linear models specifying a random effect at the health and social service center level. We started with bivariate analyses to examine the relationship between intention and the independent variables of interest (at the 0.20 alpha level). Then we performed multivariate regression analysis using a manual backward stepwise selection of the variables with a significance level (p-value) of 0.05. After obtaining the final model, we reintroduced the variables that had been excluded during the selection process one by one into the model to assess whether their inclusion improved its performance. In the final model, we considered p-values < 0.05 as statistically significant. We performed all the analyzes using R version 4.1.2. All tests were two-sided, and a p-value of < 0.05 was considered statistically significant.

## Results

### Flow of the trial and participant characteristics

**Fig. 1:**
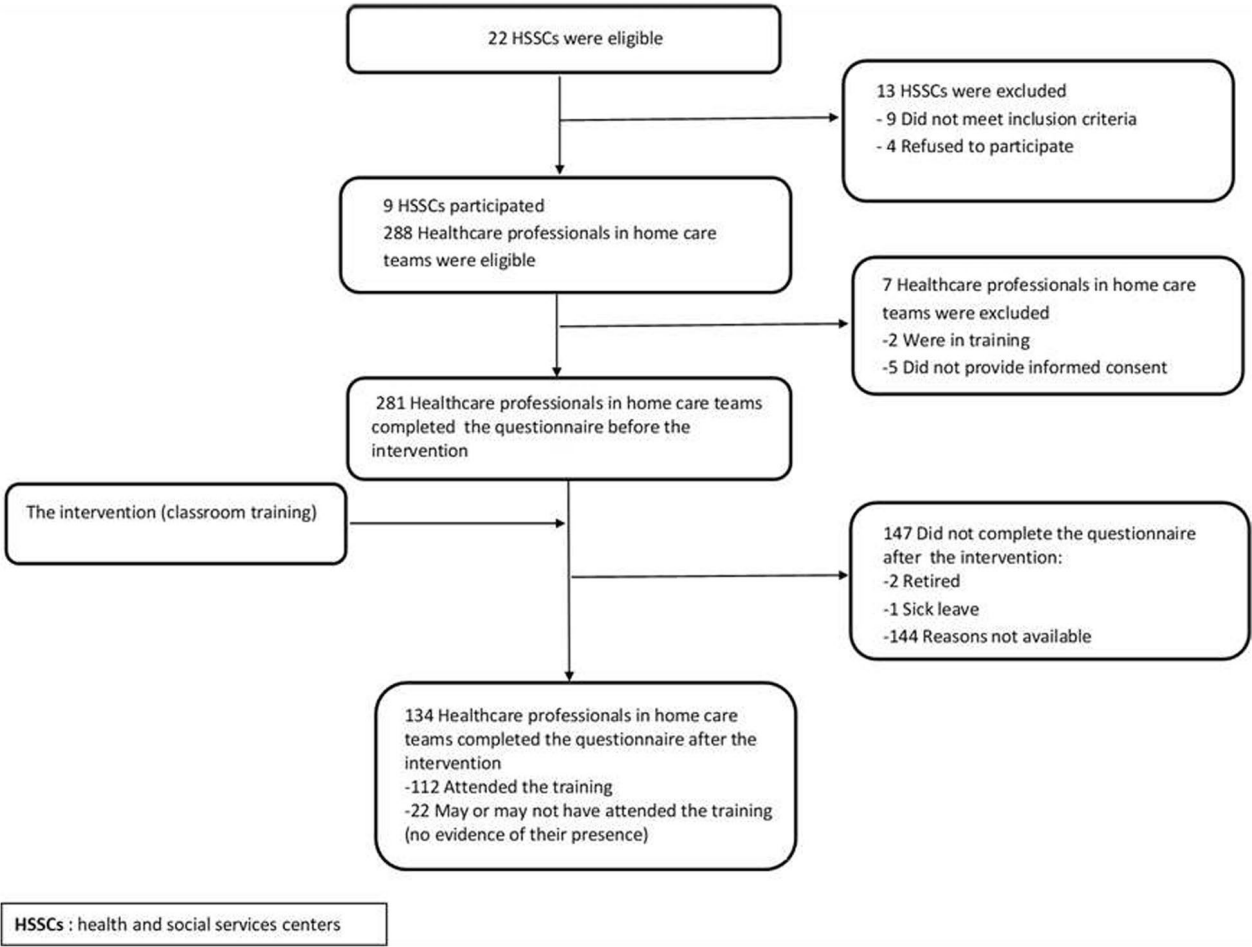
Flow chart

A total of 281 healthcare professionals in home care teams were recruited at the start but 134 provided data before and after the planned intervention and thus were included in these analyses (completed pairs of pre and post questionnaires) (Fig. 1). There were 22 health professionals who had completed both questionnaire (pre- and post-) but for whom we were not able to specify for sure if they had been exposed to the intervention as we could not find evidence of their signature on the list of trainees presence at the workshop. We thus conducted sensitivity analyses with and without them and observed no change in our results (data not shown). The characteristics of the 134 who were included in the analyses are described in Table 1. Mean (± SD) age was 42 (± 9.3) years. Mean patients served per week was 13 (± 7.6) and mean years of practice in home care was 11 (± 7.0).

**Table 1:** Socio-demographic characteristics of healthcare professionals in home care teams

Characteristics of healthcare professionals in home care teams	*n* = 134
	**Mean (± SD)**
**Age (years)**	42 (± 9.3)
**Number of clients served per week**	13 (± 7.6)
**Number of years of practice in home care**	11 (± 7)
	**N (%)**
**Sex**	
Female	122 (90.9)
Male	12 (9.1)
**Profession**	
Social workers	89 (67.1)
Nurses	25 (18.7)
Other (assistance technician, nutritionist, clinical advisor, specialized educator, etc.)	10 (7.5)
Occupational therapists	8 (6)
Missing data	2 (0.7)
**Level of education** ^b^	
Diploma to work in health and social services only	65 (48.5)
Other diploma	
Bachelor's degree	34 (25.3)
College diploma	17 (12.6)
Other (Certificate in Gerontology, Certificate in Developmental Psychology, Certificate in Management, Certificate in History, etc.)	9 (6.7)
Master's degree	7 (5.1)
High school diploma	2 (1.4)
**In person IP-SDM training**	
Yes	112 (83.6)
Not sure^a^	22 (16.4)
**Preliminary online general SDM tutorial**	
Yes	105 (78.3)
Not sure^a^	29 (21.7)

*SD* standard deviation

^a^We did not have robust evidence on whether these participants had in fact completed the preliminary online tutorial, or the in-person training, or not

^b^highest level of education obtained

Most of the healthcare professionals in home care teams were female (90.9%). Most were social workers (66.9%), and a bachelor’s degree was most frequently the highest qualification (25.3%).

### Intention before and after the intervention

Mean scores of intention to engage in IP-SDM (outcome of interest) (*n* = 134) decreased from 5.84 (± 1.19) to 5.54 (± 1.35). Beliefs about consequences also decreased from 5.89 (± 1.12) to 5.65 (± 1.19) (Table 2)**.** Similarly, mean score of social influence and belief about capabilities decreased from 5.50 (± 1.03) to 5.42 (± 1.09) and 5.58 (± 1.10) to 5.51 (± 1.15) respectively. Considering intention-to-treat [20], mean score of moral norm increased from 6.07 (± 1.02) to 6.15 (± 0.89). Wilcoxon signed ranks tests confirmed these results. We noted a significant difference only for intention and beliefs about consequences (Table 2). However, due to the scope of the difference, less than half of a standard deviation, these differences are most probably not clinically significant [21].

**Table 2:** Comparison of the intention to engage in IP-SDM of healthcare professionals in home care teams before and after the intervention (repeated measures model & Wilcoxon signed-ranks test) (*n* = 134)

Psychosocial variables (*n* = 134)	The mean score, before the intervention mean (± SD)	The mean score, after the intervention mean (± SD)	Estimates (ß) (95% CI)	*P*-value*	*P*-value**
Intention	5.84 (± 1.19)	5.54 (± 1.35)	-0.29 (-0.53; -0.05)	0.02	0.02
Beliefs about consequences	5.89 (± 1.12)	5.65 (± 1.19)	-0.24 (-0.47; -0.01)	0.03	0.03
Moral norm	6.07 (± 1.02)	6.15 (± 0.89)	0.07 (-0.11; 0.26)	0.43	0.52
Social influences	5.50 (± 1.03)	5.42 (± 1.09)	-0.08 (-0.29; 0.13)	0.46	0.68
Beliefs about capabilities	5.58 (± 1.10)	5.51 (± 1.15)	-0.07 (-0.26; 0.11)	0.42	0.80

A *p*-value < 0.05 was used as the statistical significance level

*SD* standard deviation, *CI* confidence interval

^*^Repeated measures model

^**^Wilcoxon signed-ranks test

Second, we did a sensitivity analysis: we only analyzed the healthcare professionals in home care teams whom we could be sure were exposed to the intervention based on the list of attendees who signed in (solely the in-person IP-SDM training) (*n* = 112), and still noted a significant difference, but only for intention, which decreased from 5.88 (± 1.22) to 5.57 (± 1.35) after the intervention, while there was no significant difference for the other psychosocial variables (Additional file 3).

Third, in additional analysis we did not detect a significant difference in the intention of healthcare professionals in home care teams who completed the online general SDM Tutorial before the in-person IP-SDM training (*n* = 197) compared to those who did not (*n* = 84) (Additional file 4).

### Factors associated with post-intervention intention

Factors associated with higher intention to engage in IP-SDM post-intervention were perception of approval by colleagues or significant others in the profession ("social influence") (ß = 0.34, *p* = 0.01) and perceptions of facilitators and barriers to adopting the behavior (“beliefs about capabilities”) (ß = 0.49, *p* < 0.01) (Table 3).

**Table 3:** Factors associated with healthcare professionals’ intention to use the IP-SDM after the intervention (*n* = 134)

**Variables**	**Bivariate** ^a^		**Final model** ^a^	
	**Estimate (95% CI)**	***P*** **-value**	**Estimate (95% CI)**	***P*** **-value**
**Sex**				
Female	Ref			
Male	0.06 (-0.70; 0.82)	0.88	-	-
**Profession**		**0.01**^╫^		0.13
Social workers	Ref		Ref	
Occupational therapists	-0.08 (-1.04; 0.84)	0.87	-0.05 (-0.53; 0.43)	0.84
Nurses	-0.77 (-1.36;-0.22)	0.01	-0.04 (-0.31;-0.40)	0.82
Other profession	-1.25 (-2.41;-0.35)	0.01	-0.73 (-1.25;-0.22)	0.01
**Level of education**		0.84		
Diploma to work in health and social services only	Ref			
High school diploma	1.01 (-0.75; 2.76)	0.27	-	-
College diploma	0.76 (0.03; 1.44)	0.03	-	-
Bachelor's degree	-0.10 (-0.64; 0.42)	0.70	-	-
Master's degree	0.05 (-0.99; 1.08)	0.93	-	-
Other diploma	0.21 (-0.65; 1.06)	0.64	-	-
**General SDM Tutorial**				
No	Ref			
Yes	-0.16 (-0.66; 0.34)	0.52	-	-
**Age (years)**	-0.03 (-0.05; -0.01)	**0.01**	-0.01(-0.01; 0.01)	0.78
**Number of years of practice in home care**	-0.02 (-0.05; 0.01)	**0.16**	0.01(-0.01; 0.03)	0.37
**Number of clients served per week**	0.02 (-0.01; 0.04)	**0.09**	0.01(-0.01; 0.02)	0.71
**Beliefs about consequences**	0.66 (0.51; 0.81)	** < 0.01**	0.14 (-0.03; 0.31)	0.12
**Moral norm**	0.89 (0.69; 1.11)	** < 0.01**	0.06 (-0.15; 0.27)	0.61
**Social influences**	0.76 (0.60; 0.92)	** < 0.01**	0.34 (0.17; 0.51)	**0.01**
**Beliefs about capabilities**	0.90 (0.78; 1.03)	** < 0.01**	0.49 (0.34; 0.63)	** < 0.01**
**R** ^**2**b^			72.1%	

╫Refers to the **F**-**test** statistic

“- “This variable was not kept in the final model

^a^Linear mixed regression model with adjustment for clustering

^b^Percentage of the variance in the dependent variable that is explained by the independent variables in the model

The variance of intention explained by these two factors was 72.1%.

## Discussion

In this study, we assessed healthcare professionals' intention to engage in an IP-SDM approach both before and after receiving in-person training. Additionally, we explored factors associated with this intention after the intervention. Contrary to our main hypothesis, we observed a decrease in intention following the intervention. However, given the scope of the decrease, this is most probably not clinically significant. Beliefs about consequences also decreased, whereas moral norm increased post-intervention. Despite a decline in social influence after the intervention, it remained associated with healthcare professionals' intention to engage in the IP-SDM approach. Similarly, beliefs about capabilities, which also decreased, were also associated with intention. These findings lead us to the make following observations:

First, intention to engage in IP-SDM had a statistically significant decrease after the intervention, but this decrease was most probably not clinically significant as it did not reach half of the standard deviation of the means for the intention to engage in IP-SDM before and after the intervention [21]. Our main hypothesis that could explain these findings is the fact that this trial was planned before a major reform in the health and social care system, initiated by the Quebec government in March 2015 [22, 23]. The reform occurred between the intervention and data collection at exit, which varied from 10 to 32 months [7]. Briefly, existing healthcare organizations were merged into 22 megastructures which took over the mandates and missions of the previous structures in their areas of jurisdiction. The new organizations gathered a much broader scope of health services under a single governing body per territory. Under this imposed merger, many healthcare teams, including home care teams, faced loss of staff, heavy workloads, low morale, and changes in team composition. This may have been behind the decrease. The ones who remained may have been simply overwhelmed trying to meet the essential basic daily needs of their clients. On the other hand, our finding might underscore a critical point: while training programs targeting behavioral changes among healthcare professionals are valuable, structural and organizational barriers within healthcare systems can impede the successful implementation of evidence-based practices such as SDM. As highlighted by Müller et al., organizational culture, leadership support, and alterations in workflow structures are pivotal factors for the effective integration of SDM in healthcare[24]. Moreover, there is a dearth of knowledge regarding the influence of system-level characteristics on SDM implementation [25]. Therefore, future research aimed at implementing IP-SDM in home-care settings should delve into the organizational and system-level characteristics that both facilitate and hinder implementation. Quantifying the varying impacts of these characteristics, understanding their potential interactions, and exploring how the system could operate differently are crucial aspects to consider in advancing SDM implementation efforts [25] and should be a target of future studies.

Second, following the training, there was a statistically significant decrease in the "beliefs about consequences" construct, which assesses perceptions of positive or negative outcomes linked to specific behaviors. Again, the scope of the decrease is most probably not clinically significant. Existing literature also suggests that some allied health professionals express frustration with engaging in SDM, citing issues such as client characteristics and misalignment with their professional frameworks [26, 27]. Our findings confirm this ambivalence among health professionals: despite recognizing the moral importance of an IP-SDM approach, the barriers within their practices and professional frameworks may block their intention to engage in IP-SDM, even after training. Future studies should address these challenges and design interventions tailored to the specific context barriers encountered by these professionals in their daily practices.

Third, factors associated with intention to engage in IP-SDM post-intervention were perception of approval by colleagues or significant others in the profession ("social influence") (ß = 0.34, *p* = 0.01) and perceptions of facilitators and barriers to adopting the behavior (“beliefs about capabilities”) (ß = 0.49, *p* < 0.01). The "social influences" construct reflects individuals' perceptions of approval or disapproval from peers regarding a specific behavior. Similarly, a separate review of 19 studies identified the key characteristics of interprofessional teams that influence implementation and that also play a role in social influence, including governance structures, communication power dynamics, and training [28]. Our intervention addressed only one of these elements – training – and it is possible that integrating these other factors into the IP-SDM model could enhance social influence. This is crucial because our data indicated a statistically significant association between social influence and intention. Consequently, future adaptations of the IP-SDM model may benefit from implementing behavior change techniques specifically targeting social influence. In a separate study with interprofessional teams within a Quebec mental health network, strategies such as providing information about peer approval, promoting trust through social comparison, and fostering social support/change emerged as relevant approaches that could be integrated in future adaptations [29, 30]. Moreover, we found a statistically significant association between beliefs about capabilities and intention. This aligns with findings from a systematic review of SDM training programs[31], which revealed that studies involving allied health professionals or nurses often indicated a desire for more training to enhance their SDM skills [32, 33]. Future interventions targeting allied healthcare professionals should consider this and incorporate additional training sessions, practice opportunities or ongoing support programs to build their beliefs about their capabilities and confidence in implementing IP-SDM effectively.

Finally, we did not detect a significant difference in intention, or in any of its variables, among healthcare professionals who attended the online training on general SDM principles meant as a prompt (Additional file 4). This suggests that the online portion of the training did not have a great impact on the predisposition of health professionals to attend the IP-SDM in-person training and thus on their intention to engage in an IP-SDM approach. This may be explained by the fact that for health professionals to invest time in training they need to feel that the material is relevant to them from start to finish. Anecdotally, participants told the research team that the online module was too general and not relevant to their work (data not shown). Thus it will be important to develop more targeted online training material in future studies [34].

The strength of this study lies in its use of a socio-cognitive theory to identify factors associated with professionals' intention to engage in an IP-SDM approach. In keeping with this theory [9], factors associated with healthcare professionals' intention to adopt a clinical practice or not were identified following a comprehensive and rigorous review. The factors identified on the basis of this theory explained more than two-thirds of the variance in intention, and our results thus confirm the theory’s hypothesis: that modifiable psychosocial factors are more likely to explain healthcare professionals' behavior change than sociodemographic characteristics. Also, a validated tool with acceptable internal consistency of constructs [19] was used to measure intention as well as its psychosocial determinants.

This study has a number of limitations. First, few healthcare professionals in home care teams completed both the pre and post intervention questionnaires, due to the major healthcare reform that took place during the study. Different information and selection biases could have occurred, as the 134 included participants may have had similar behavioral intentions, and different from the 147 who did not complete the questionnaire after the intervention. Second, because we used a self-reported questionnaire, a social desirability bias may apply. In other words, a healthcare professional may have indicated a higher intention than her actual intention to satisfy a certain social desirability [35].

## Conclusion

We found that the level of intention of healthcare professionals to engage in the IP-SDM approach decreased after a training session on the IP-SDM approach and the use of a PtDA. Based on our results, IP-SDM training should use behavior change techniques that focus on social influence and beliefs about capabilities. Designers of training interventions should also focus on strategies to withstand or mitigate disruptions at the system and organizational levels, thus favoring sustainability. Further research could also explore the effect of more training, as the initial training introduced the IP-SDM concept but then revealed the providers' lack of readiness to engage in SDM.

The results of this study will enable health system jurisdictions to plan more effective training of healthcare professionals to engage in the IP-SDM approach and, to a certain extent, better understand how major healthcare reform can hamper implementation of desirable clinical behaviors.

### Supplementary Information

**Supplementary Material 1**.

**Supplementary Material 2**.

**Supplementary Material 3**.

**Supplementary Material 4**.

## Data Availability

The datasets used and/or analyzed during the current study are available from the corresponding author on reasonable request.

## Abbreviations

CPD-Reaction: Continuing Professional Development-Reaction
HSSCs: Health and social services centers
PtDA: Patient decision aid
IP-SDM: Interprofessional shared decision-making
