# Supplementary figures and images for: Changes in intention to use an interprofessional approach to decision-making following training: a cluster before-and-after study
Source: BMC Health Serv Res. 2024 Apr 8;24:437. doi: 10.1186/s12913-024-10899-z (PMC11000315; doi:10.1186/s12913-024-10899-z)

## Additional file 1 : Detailed flow chart

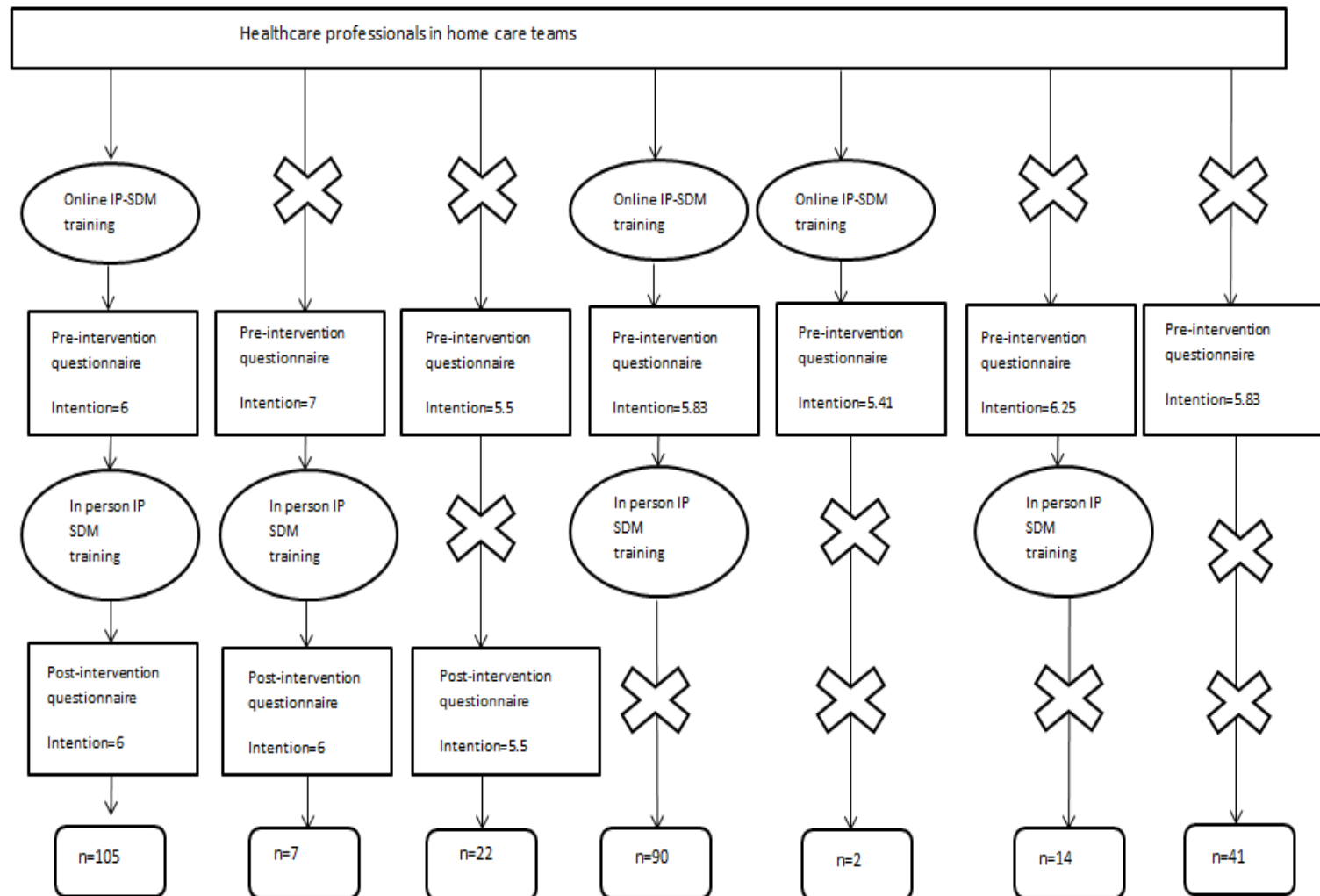

Supplement: Supplementary file 1 — Supplementary Material 1. [file 12913_2024_10899_MOESM1_ESM.pdf]
