# Supplementary material for: Changes in intention to use an interprofessional approach to decision-making following training: a cluster before-and-after study
Source: BMC Health Serv Res. 2024 Apr 8;24:437. doi: 10.1186/s12913-024-10899-z (PMC11000315; doi:10.1186/s12913-024-10899-z)
Supplement: Supplementary file 2 — Supplementary Material 2. [file 12913_2024_10899_MOESM2_ESM.pdf]

Additional file 2: Ottawa Decision Support Model[16].

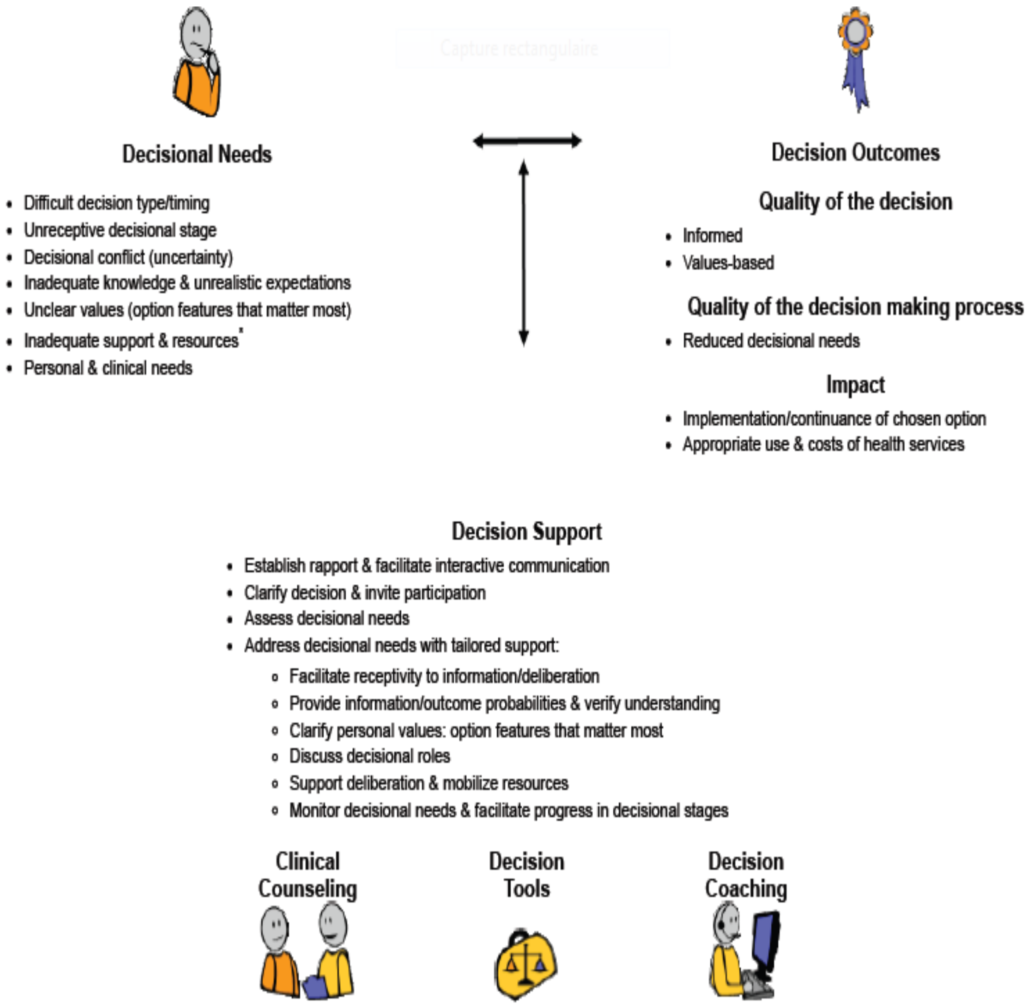

Legend : © 2020 Stacey, Legare, Boland, Lewis, Loiselle, Hoefei, Garvelini, O Cornor**.**
