# Supplementary material for: Changes in intention to use an interprofessional approach to decision-making following training: a cluster before-and-after study
Source: BMC Health Serv Res. 2024 Apr 8;24:437. doi: 10.1186/s12913-024-10899-z (PMC11000315; doi:10.1186/s12913-024-10899-z)
Supplement: Supplementary file 3 — Supplementary Material 3. [file 12913_2024_10899_MOESM3_ESM.pdf]

Additional file 3: Comparison of the intention of healthcare professionals who attended the in-person class before and after the intervention (repeated measures model & Wilcoxon signed-ranks test) (n=112)

**Psychosocial variables (n=112)**	The mean score, **after the intervention** mean (± SD)	The mean score, **before the intervention** mean (± SD)	**estimates (ß) (95% CI)**	**P-value***	**P-value****
**Intention**	5.57 (±1.35)	5.88 (±1.22)	-0.31 (-0.56; -0.06)	**0.01**	**0.01**
**Beliefs about consequences**	5.66 (±1.19)	5.87 (±1.15)	-0.20 (-0.46;0.05)	0.12	0.14
**Moral norm**	6.17 (±0.88)	6.09 (±1.07)	0.07 (-0.13 ; 0.28)	0.47	0.58
**Social influences**	5.39 (±1.11)	5.51 (±1.04)	-0.13 (-0.36 ; 0.10)	0.28	0.38
**Beliefs about capabilities**	5.53 (±1.12)	5.59 (±1.14)	-0.05 (-0.25 ; 0.13)	0.56	0.79

SD: standard deviation

A p-value <0.05 was used as the statistical significance level

*Repeated measures model

**Wilcoxon signed-ranks test
