# Supplementary material for: Changes in intention to use an interprofessional approach to decision-making following training: a cluster before-and-after study
Source: BMC Health Serv Res. 2024 Apr 8;24:437. doi: 10.1186/s12913-024-10899-z (PMC11000315; doi:10.1186/s12913-024-10899-z)
Supplement: Supplementary file 4 — Supplementary Material 4. [file 12913_2024_10899_MOESM4_ESM.pdf]

Additional file 4: Comparison of the intention of healthcare professionals who attended the online training with healthcare professionals who did not attend the online training

Psychosocial variables	The mean score, for professionals who attended the online training (n=197)	The mean score, for professionals who did not attend the online training (n=84)	estimates (ß) (95% CI)	P-value**
**Intention**	5.81 (±1.15)	5.75 (±1.01)	0.06 (-0.04; 0.49)	0.34
**Beliefs about consequences**	5.88 (±1.09)	5.85 (±1.06)	0.03 (-0.08 ; 0.48)	0.71
**Moral norm**	6.09 (±0.97)	6.06 (±0.90)	0.03 (-0.09 ; 0.3)	0.60
**Social influences**	5.54 (±0.98)	5.36 (±0.97)	0.18 (-0.03 ; 0.46)	0.12
**Beliefs about capabilities**	5.6 (±1.04)	5.62 (±0.93)	-0.01 (-0.29 ; 0.29)	0.96

SD: standard deviation
A p-value <0.05 was used as the statistical significance level
**Wilcoxon signed-ranks test
